# Exploring Genetic Interactions with Telomere Protection Gene *pot1* in Fission Yeast

**DOI:** 10.3390/biom13020370

**Published:** 2023-02-15

**Authors:** Masaru Ueno

**Affiliations:** 1 Graduate School of Integrated Sciences for Life, Hiroshima University, Higashi-Hiroshima 739-8530, Japan; scmueno@hiroshima-u.ac.jp; Tel.: +81-82-424-7768; 2 Hiroshima Research Center for Healthy Aging (HiHA), Hiroshima University, Higashi-Hiroshima 739-8530, Japan

**Keywords:** telomere, ring chromosome, DNA repair, chromosome segregation

## Abstract

The regulation of telomere length has a significant impact on cancer risk and aging in humans. Circular chromosomes are found in humans and are often unstable during mitosis, resulting in genome instability. Some types of cancer have a high frequency of a circular chromosome. Fission yeast is a good model for studying the formation and stability of circular chromosomes as deletion of *pot1* (encoding a telomere protection protein) results in rapid telomere degradation and chromosome fusion. Pot1 binds to single-stranded telomere DNA and is conserved from fission yeast to humans. Loss of *pot1* leads to viable strains in which all three fission yeast chromosomes become circular. In this review, I will introduce *pot1* genetic interactions as these inform on processes such as the degradation of uncapped telomeres, chromosome fusion, and maintenance of circular chromosomes. Therefore, exploring genes that genetically interact with *pot1* contributes to finding new genes and/or new functions of genes related to the maintenance of telomeres and/or circular chromosomes.

## 1. Introduction

**Figure 1:**
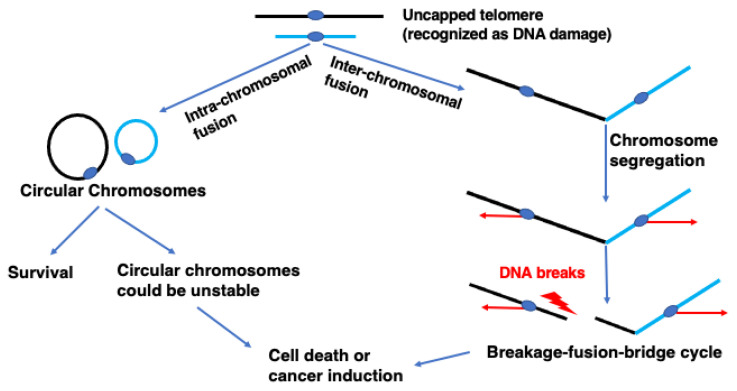
Uncapping of telomeres leads to chromosome abnormalities. Telomere uncapping results in breakage–fusion–bridge (BFB) cycles or chromosome circularization. Inter-chromosomal fusion generates dicentric chromosomes. Segregation of the two centromeres towards opposite poles creates chromatin bridges, which will result in DSB. The DSB ends will be fused, generating dicentric chromosomes. This breakage–fusion–bridge (BFB) cycle results in translocations and aneuploid nuclei, which can drive cancer. Intra-chromosomal fusion generates circular chromosomes, which can be unstable, and result in chromosome breaks and/or rearrangement that could induce cancer.

Telomeres cap the end of the chromosome to maintain genome stability [1,2]. They consist of a protein complex called Shelterin, which binds the end of the chromosome [3]. Telomere DNA consists of tandem nucleic acid repeats (TTAGGG) in vertebrates. Repeats of telomere DNA shorten with each cell division in most normal cells, except stem and germ cells. Telomere DNA can be elongated by telomerase, a ribonucleoprotein reverse transcriptase that is active in most cancer cells but inactive in most normal cells. When telomere DNA becomes short and uncapped in normal cells, cells stop dividing through DNA damage checkpoint activation [4]. However, if cells fail to stop cell division due to defects in checkpoint activation, telomere DNA becomes critically short and becomes a substrate for homologous recombination (HR) and/or non-homologous end joining (NHEJ) [5]. HR activity in uncapped telomeres can maintain telomeres without telomerase activity in a process called alternative lengthening of telomeres (ALT), found in ~15% of cancer cells [6]. NHEJ activity in uncapped telomeres results in telomere fusion between the ends of inter-chromosomes or between those of intra-chromosomes (Figure 1). Telomere fusion between inter-chromosome ends results in a dicentric chromosome that forms an anaphase bridge in mitosis. Anaphase bridge breakage results in a DNA double-strand break, which can lead to ectopic fusion and result in another dicentric chromosome. These breakage–fusion–bridge events result in chromosomal instability, which promotes cancer development and telomerase activation [4]. 

Telomere fusion between intra-chromosome ends results in a circular chromosome. A circular chromosome, also called a ring chromosome, can be found in human cells, although their frequency is very low (approximately 1:50,000) [7]. Many human carriers of a ring chromosome have ring chromosome syndrome, which is characterized by short stature and developmental delay [8]. A ring chromosome induces many secondary chromosome rearrangements, resulting in an increased risk of cancer due to a loss of cancer suppressor genes or other genetic changes. Stabilizing the ring chromosome could reduce the risk of cancer in carriers. A ring chromosome can also be found at a high frequency in some types of cancer cell. In some mesenchymal origin tumors, more than 70% of cells could have a ring chromosome [9]. Therefore, the ring chromosome could be a potential target for cancer therapy. However, the nature of the ring chromosome in humans has not been well studied because of the lack of a model system with which to do so.

## 2. Telomere Protection by Pot1 in Human and in Fission Yeast *Schizosaccharomyces pombe*, and by Cdc13 in Budding Yeast *Saccharomyces cerevisiae*

Human POT1, a component of Shelterin complex, binds to single-stranded telomere DNA [10,11,12]. POT1 forms a subcomplex with TPP1, which recruits telomerase to telomere [13,14]. POT1 knock down in normal human diploid fibroblasts (IMR-90) induces apoptosis, senescence, or telomere fusion, showing that POT1 inhibits the DNA damage response at telomeres [15]. As POT1 blocks the access of telomerase to telomere DNA by the covering of the G-strand overhang, human POT1 acts a negative regulator of telomerase [14,16,17]. Indeed, mutation in POT1 results in telomere elongation [18,19,20]. 

Fission yeast is an excellent model organism for studying telomere maintenance and chromosomal instability induced by telomere dysfunction. The Shelterin complex is similar to that of mammalian cells. For example, the POT1 ortholog, also called Pot1 in *Schizosaccharomyces pombe*, binds to single-stranded telomere DNA and prevents the DNA damage response [10,21]. *S. pombe* Tpz1, the TPP1 ortholog, forms a complex with Pot1, and this complex recruits telomerase through Ccq1 [22]. As expression of N-terminal truncated Pot1 results in telomere elongation, *S. pombe* Pot1 is also thought to function as a negative regulator of telomerase [23]. *trt1* encodes a catalytic subunit of telomerase in *S. pombe* [24]. Telomerase is always active in *S. pombe*, similar to human cancer cells that have telomerase activity. The deletion of *trt1* results in gradual telomere shortening during several cell divisions [24]. Survivors of the *trt1* knockout have either three circular chromosomes or three linear chromosomes, in which telomeres are maintained by HR, similar to ALT cells in human cancer [25]. 

Circularization of one chromosome has been reported in many organisms, including humans, plants, and *S. cerevisiae* [7,26,27,28,29]. However, the chance of the circularization of all chromosomes is very low if the chromosome number is not low. *S. pombe* is a unique organism in that there is a high chance of circularization of all three chromosomes. Unlike *trt1* knockout, the deletion of *pot1* results in rapid telomere shortening and three circular chromosomes in the survivors [10]. It remains unclear why deletion of *pot1* does not result in survivors maintaining telomeres by HR. One possible reason is that extensive degradation of chromosome ends results in the complete loss of template DNA, which is necessary for HR at telomeres. Chromosome fusion in the *pot1* knockout is mediated by single-strand annealing (SSA) [30]. Both ends of chromosomes I and II have regions of extensive homology, which are used for SSA when *pot1* is deleted. Interestingly, knockout of genes required for SSA, such as *rad22* (fission yeast homologs of Rad52), *srs2*, *rad16* (ERCC1/XPF endonuclease), and *lig4* are synthetically lethal with *pot1* deletion. Both ends of chromosome III have long arrays of rDNA repeats adjacent to telomere repeats but lack regions of extensive homology, which are used for SSA. The mechanism of chromosome III circularization in the *pot1* disruptant remains unclear. A *rad3 tel1* (homologues of ATR and ATM, respectively) double mutant with three circular chromosomes contains microhomologies of three and thirteen nucleotides at the fusion site in the rDNA region of chromosome III, implying that the mechanism of chromosome fusion in chromosome III is different from that in other chromosomes [31]. Degradation of 5′-single-stranded DNA is required for chromosome fusion by SSA in uncapped telomeres. Double deletion of *rqh1* (human BLM/*S. cerevisiae SGS1*) and *exo1* slows down the degradation of 5′-single-stranded DNA of the telomere end after *pot1* shut-off, suggesting that Rqh1 and Exo1 play redundant roles in the degradation of uncapped telomeres [32]. Deletion of *rqh1* and/or *exo1* suppresses acute growth defect induced by *pot1* shut-off, further supporting this idea [32]. However, the chromosome ends in the *pot1 rqh1 exo1* triple mutant were thought to be fused by SSA, implying that unknown nucleases can resect uncapped telomeres in the absence of both Exo1 and Rqh1 [33].

Although there is no clear sequence homology, the functional counterpart of *S. pombe* Pot1 is Cdc13 in *S. cerevisiae* [34]. Cdc13 forms a complex with Stn1 and Ten1 and this binds to the single-stranded telomeric DNA [35]. Inactivation of Cdc13 using the *cdc13-1* ts mutant results in degradation of telomeric DNA and DNA damage checkpoint dependent cell cycle arrest [36]. The nucleases responsible for the degradation in the *cdc13-1* ts mutant are Exo1 and the Pif1 helicase with an unknown nuclease [37]. A common function of human POT1, *S. pombe* Pot1, and *S. cerevisiae* Cdc13 is the protection of telomere overhang from DNA damage checkpoint activation. Unlike *S. pombe* and *S. cerevisiae*, knock down of human POT1 does not result in rapid telomere degradation. This may be due to high NHEJ preference in humans and high HR preference in *S. pombe* and *S. cerevisiae*, which would require high nuclease activity for HR or SSA initiation. 

## 3. Lethality of *pot1 rqh1* Double Mutant Is Suppressed by Inactivation of Homologous Recombination

**Figure 2:**
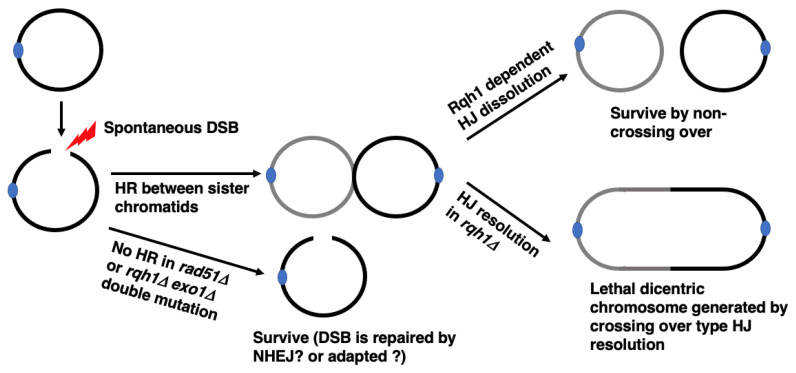
Consequence of HR in cells with circular chromosomes in the absence or presence of Rqh1. Spontaneous DSB in a circular chromosome would result in Exo1 and/or Rqh1 dependent DSB end processing and Rad51 dependent generation of recombination intermediates between sister chromatids. This intermediate can be resolved without crossing over by Rqh1-Top3 dependent double Holliday junction (HJ) dissolution. In the absence of Rqh1, this intermediate will be resolved by nuclease(s), which could generate dicentric circular chromosome dimer by crossing over. Even if spontaneous DSB in a circular chromosome is not repaired by HR due to the defect in HR activity, this DSB would not result in cell death, possibly due to adaptation or to NHEJ dependent DSB repair.

*S. pombe* Rqh1 is a conserved RecQ helicase involved in several steps of HR [38]. Deletion of *rqh1* results in hyperrecombination and the accumulation of recombination intermediates [39,40]. These results and those of other studies suggest that Rqh1 inhibits strand invasion and promotes the resolution of recombination intermediates. Moreover, it has been suggested that Rqh1 is involved in the resection of DNA Double Strand Break (DSB) ends [32,33,41,42]. *pot1* and *rqh1* are synthetically lethal [30], for two possible reasons, the first being that the chromosome cannot be fused due to defects in resection and SSA [30]. The second reason is that circular chromosomes are not maintained. The chromosome fusion is detected after *pot1* shut-off in the *rqh1*-deletion background, ruling out the first possibility regarding chromosome fusion [33]. To test the second possibility (that the circular chromosomes cannot be maintained in the absence of Rqh1), we screened genes that suppress the lethality of the *pot1 rqh1* double mutant. We found that its lethality was suppressed by the deletion of *rad51*, which plays a central role in HR [33]. The lethality of the *pot1 rqh1* double mutant was also suppressed by deletion of *exo1*, which is thought to play a redundant role with Rqh1 in the resection of DNA DSB ends that is essential for HR initiation [32,33,41,42]. Therefore, it is likely that HR activity was completely blocked in the *rqh1 exo1* double mutant (Figure 2). These results suggest that the lethality of *pot1 rqh1* is suppressed by HR inactivation. Rqh1 inhibits crossing over, which can produce circular dimers with two centromeres when a DSB in circular chromosomes is repaired by HR between sister chromatids. The shut-off of *rqh1* in the *pot1* disruptant, which has a circular chromosome, results in chromosome segregation defects. Considered together, we concluded that Rqh1 is required for the maintenance of circular chromosomes by inhibiting the production of circular dimers (Figure 2). 

Top3 functions with Rqh1 to inhibit crossover by dissolution of double Holliday junctions (HJ) [43], suggesting that Top3 is required for the maintenance of circular chromosomes by inhibiting the production of circular dimers. *top3* is an essential gene in *S. pombe*, but the *top3 rqh1*-helicase dead (*rqh1-hd*) double mutant is viable [44]. As discussed later, the *pot1 rqh1-hd* double mutant is also viable [45]. We found that the *pot1 rqh1-hd top3* triple mutant was lethal, suggesting that Top3 is required for the maintenance of circular chromosomes. Interestingly, the lethality of the *pot1 rqh1-hd top3* triple mutant was also suppressed by *rad51* deletion [33]. These facts further suggest that the inhibition of crossover is critical for maintaining circular chromosomes. 

## 4. *pot1 rqh1-hd* Cells Maintain Telomeres by HR and Accumulate Recombination Intermediates at Telomeres

The *pot1 rqh1* double mutant is lethal, whereas the *pot1 rqh1-hd* double mutant is not [45]. This is surprising because the helicase activity of Rqh1 is thought to constitute its main function [46]. To study why the *pot1 rqh1-hd* double mutant was not lethal, we characterized the phenotype of the *pot1 rqh1-hd* double mutant. The *pot1 rqh1-hd* double mutant had a stronger subtelomere signal than wild-type cells, suggesting that chromosome ends are maintained by HR using subtelomeric DNA as a template [45]. This subtelomere signal is completely abolished through the deletion of *rad51*, further suggesting that chromosome ends are maintained by HR in the *pot1 rqh1-hd* double mutant. Next, we analyzed chromosome ends using PFGE to confirm that the chromosomes of the *pot1 rqh1-hd* double mutant were linear. We could not detect any bands corresponding to the chromosome end fragments using PFGE. DNA with branched structures such as recombination intermediates cannot enter a pulsed-field gel. As Rqh1 is required for the resolution of recombination intermediates [40], these facts suggest that recombination intermediates accumulate at the chromosome ends in the *pot1 rqh1-hd* double mutant. It remains unclear why the *pot1 rqh1 null* double mutant cannot maintain telomeres by HR. I assume that the Rqh1-hd protein may prevent nuclease-dependent rapid telomere degradation as seen in the *pot1* disruptant, which could increase the chance of HR-dependent telomere maintenance.

The phenotype of the *pot1 rqh1-hd* double mutant was unique compared to that of the *trt1* disruptant that maintains telomeres by HR, because the recombination intermediates do not accumulate at the telomere in the *trt1* disruptant. The *pot1 rqh1-hd* double mutant had defects in chromosome segregation and was sensitive to the anti-microtubule drug thiabendazole (TBZ) [45]. These phenotypes can be explained as follows: the presence of recombination intermediates at telomeres prevents chromosome segregation. Inhibition in mitotic spindles by TBZ increases chromosome segregation defect in the *pot1 rqh1-hd* double mutant. TBZ sensitivity of *pot1 rqh1-hd* double mutant is suppressed by mutation in *chk1*, *cdc2-3w*, or *wee1 mik1* double mutation [47,48]. All of these mutations shorten G2 phase length, suggesting that long G2 accumulates recombination intermediates in *pot1 rqh1-hd* double mutant that make cells sensitive to TBZ. 

Surprisingly, the spindle checkpoint was activated in the *pot1 rqh1-hd* double mutant [47]. The spindle checkpoint monitors defects in kinetochore–microtubule attachment or lack of tension at the kinetochores. Therefore, our results suggest that accumulation of recombination intermediates at the telomere (or elsewhere) somehow induces defects in kinetochore–microtubule attachment or a lack of tension at the kinetochores. Indeed, the growth of the *pot1 rqh1-hd* double mutant is made worse by the deletion of gene encoding spindle checkpoint proteins Mad2 or Bub1 [47]. Moreover, Mad2 foci persisted longer than usual at kinetochores in the double mutant, suggesting a defect in kinetochore–microtubule attachment in the *pot1 rqh1-hd* double mutant. It remains unclear how the accumulation of recombination intermediates at the telomere induces defects in kinetochore-microtubule attachment. 

Centromeric adaptor, shugoshin Sgo2, mainly binds to subtelomeres during interphase, but binds to centromeres during mitosis [49], implying that the function of proteins that localize to both telomeres and centromeres may be affected in the *pot1 rqh1* double mutant. CENP–S and CENP–X, also called Mhf1 and Mhf2, respectively, localize to the centromere and play a role in recombination and repair [50]. This implies that the function of proteins that localize to both recombination sites and centromeres may be affected in the *pot1 rqh1-hd* double mutant. Further investigation will reveal the mechanism by which the accumulation of recombination intermediates at the telomere induces defects in kinetochore–microtubule attachment. 

## 5. *pot1* and Genes Encoding the Chromosomal Passenger Complex (CPC) Are Lethal

The chromosomal passenger complex (CPC) comprises Aurora-B protein kinase, the inner centromere protein INCENP, survivin, and borealin. The CPC plays a crucial role in mitosis [51]. The *S. pombe* CPC consists of the aurora-related kinase Ark1, survivin homolog Bir1, and inner centromere protein Pic1 [52]. The *S. pombe* CPC is important for proper bipolar attachment of sister chromatids during metaphase [53]. Interestingly, we found that genes encoding the CPC, *ark1*, *bir1*, and *pic1* genetically interact with *pot1* in fission yeast [54]. *ark1, bir1*, and *pic1* are essential for growth but temperature-sensitive (*ts*) mutants can grow at a semi-permissive temperature. We found that the double mutants of *pot1* and *ark1* or *bir1* or *pic1 ts* alleles were synthetically lethal at semi-permissive temperatures [54]. The *pot1 pic1* double mutant, which has circular chromosomes, is viable at a permissive temperature (25 °C). This strain lost viability completely at a semi-permissive temperature (33 °C), whereas the *pic1* single mutant could survive at 33 °C. These facts suggest that Pic1 and most likely the CPC are required for the growth of strains that have circular chromosomes. The *trt1 pic1* double mutant, which has circular chromosomes at a permissive temperature (25 °C), showed the same phenotype as the *pot1 pic1* double mutant, further demonstrating that the CPC is required for the growth of strains with circular chromosomes. 

Sgo2 is required for centromeric localization of the Aurora kinase complex [55]. However, the *sgo2 pot1* double mutant is not synthetically lethal [54]. Therefore, the function of CPC, which is not related to Sgo2 function, is important for the growth of cells with circular chromosomes. The *pot1 pic1* double mutant displayed elevated rates of chromosome segregation defects at the permissive temperature (33 °C), suggesting that the role of the CPC in chromosome segregation is important for the growth of cells with circular chromosomes. One possible candidate for this function is condensin, because the condensin subunit Cnd2 is phosphorylated by the CPC subunit Ark1 throughout mitosis [56]. Circular chromosomes may have difficulty with chromosome condensation because they are topologically constrained. Identifying the target of Ark1, which is essential for the growth of cells that have circular chromosomes, will contribute to the understanding of why the *pot1 cpc* complex is synthetically lethal. 

We also found interesting genetic interaction between *pot1* and *sgo2* and *swi6* [54]. Both *pot1 sgo2* double mutant and *pot1 swi6* double mutant show higher chromosome segregation defects than each single mutant [54]. Both Sgo2 and Swi6 localize to centromere and (sub) telomere [57], implying that telomere loss may affect centromere function. Defect of *dcr1* disruptant in pericentric heterochromatin silencing is suppressed by deletion of *pot1*, further suggesting that telomere loss may affect centromere function [58]. 

## 6. Histone H4 Acetylation and the Bromodomain Protein Bdf2 Are Required for the Growth of Cells with Circular Chromosomes

A ring chromosome is a cytogenetic hallmark of atypical lipomatous tumors and can be used to determine a proper diagnosis [59]. Chemicals that inhibit growth of cells that have circular chromosomes can be used as anti-cancer drugs for cancers in which most cells have a circular chromosome, such as dermatofibrosarcoma protuberans (70%) and atypical lipomatous tumors (85%) [60]. A chemical that inhibits the growth of fission yeast *pot1* disruptant with circular chromosomes could be a candidate for this purpose. We found that (+)-JQ1, which is a potent, high-affinity, selective BET bromodomain inhibitor, inhibits the growth of fission yeast *pot1* disruptant more than the control strain with linear chromosomes [61]. (+)-JQ1 displaces BRD4 from chromatin [62]. Human BRD4 binds to acetylated lysine in histone tails via a bromodomain [63]. The orthologs of human BRD4 are Bfd1 and Bfd2 in fission yeast. Therefore, one possible explanation for the growth inhibition of *pot1* disruptant by (+)-JQ1 could be that (+)-JQ1 inhibits fission yeast Bfd1 and/or Bfd2, and fission yeast *bfd1* and/or *bfd2* are important for the growth of *pot1* disruptants with circular chromosomes. *pot1 bdf2*, but not *pot1 bdf1*, was found to be synthetically lethal [61]. These results suggest that (+)-JQ1 inhibits fission yeast Bdf2. To study why the *pot1 bdf2* double mutant is synthetically lethal, we created a *bdf2-ts* mutant that loses Bdf2 function at a high temperature (36 °C). The *pot1 bdf2-ts* double mutant with circular chromosomes is viable at a permissive temperature (25 °C) but shows a synthetic growth defect at 36 °C. These results demonstrated that Bdf2 plays an important role in the growth of cells with circular chromosomes. 

*mst1* encodes a histone acetyltransferase in fission yeast. Both Bdf2 and Mst1 are required for maintaining proper histone H4 acetylation level, suggesting functional overlap between Bdf2 and Mst1 [64,65]. Because Mst1 is essential for growth, we used the *mst1-ts* mutant [66]. We found that the *pot1 mst1-ts* double mutant, which has circular chromosomes at a permissive temperature (25 °C), showed a synthetic growth defect at a semi-permissive temperature (31 °C). These results suggested that maintaining proper histone H4 acetylation levels is important for the growth of cells with circular chromosomes. The change in histone H4 acetylation levels in *bdf2* and *mst1* mutants could affect whole-genome gene expression profiling. Therefore, analysis of changes in gene expression profiling in the *bdf2* and *mst1* mutants would improve our understanding of the importance of Bdf2 and Mst1 for the growth of cells that have circular chromosomes. 

## 7. *pot1* and Gene Encoding Phosphatidylinositol 4-Kinase Pik1 Is Lethal

Genes that genetically interact with *pot1* can be screened using forward genetics. We found that a mutation in gene encoding phosphatidylinositol 4-kinase Pik1, called *pik1-1*, is synthetically lethal with *pot1* [67,68]. Pik1 localizes to the Golgi and phosphorylates PI to produce PI(4)P [69]. The shut-off of *pot1* in the *pik1-1* mutant resulted in chromosome fusion, demonstrating that Pik1 is not required for chromosome circularization, suggesting that Pik1 is required for the growth of cells with circular chromosomes. 

We found that the PI(4)P level in the Golgi was reduced in *pik1-1*, suggesting that PI(4)P in the Golgi is required for the growth of the *pot1* disruptant. However, it has been argued that PI(4)P has a nuclear role in humans [70]. Moreover, *S. cerevisiae* Pik1 localizes to the nucleus in addition to the Golgi apparatus [71]. Therefore, although it remains unclear whether *S. pombe* Pik1 localizes to the nucleus [72], *S. pombe* Pik1 may function to regulate PI(4)P levels in the nucleus and/or in the nuclear membrane, and this may contribute to the growth of cells with circular chromosomes. It is also possible that the *pik1* mutation may affect the sorting of proteins necessary for the maintenance of chromosome circularization due to defects in Golgi functions. 

## 8. The Effect of Chromosome Circularization in Torsional Constraint, DNA Damage, and Chromosome Organization 

DNA replication and transcription generate a DNA topological strain that can inhibit DNA replication and transcription if the DNA topological strain is not removed [73]. Excessive supercoiling is mitigated by topoisomerases (I and II). In *S. cerevisiae*, DNA is not torsionally constrained at the chromosomal ends, making it difficult to evaluate the impact of torsional constraints when the chromosome is linear [74]. The circular chromosome provides an excellent model system to study torsional constraints because the chromosome ends are physically fused. Cells with circular chromosomes are highly sensitive to the topoisomerase I inhibitor, camptothecin CPT [61,75]. We found that DNA topoisomerase II poisons, such as mitoxantrone and its analog, inhibit the growth of cells with circular chromosomes more efficiently than cells with linear chromosomes (manuscript in preparation). These results demonstrate that cells with circular chromosomes are useful for studying the importance of torsional constraint and screening novel topoisomerase inhibitors and proteins that are involved in the regulation of torsional constraint. 

Five percent of the *pot1* disruptants have RPA foci (a marker of DNA damage), compared to ~two percent of wild-type cells, suggesting that circular chromosomes have more risk of spontaneous DNA damage [76]. This could be because of the higher torsional constraint in the *pot1* disruptant. Consistently, the *pot1* disruptant is more sensitive to the alkylating agent methyl-methanesulfonate MMS than the wild-type strain [77]. The Rad9-Rad1-Hus1 (9-1-1) complex involved in DNA damage checkpoint is loaded onto chromatin when DNA replication is halted [78]. We found that double mutants between *pot1* and genes encoding the 9-1-1 checkpoint complex (Rad9-Rad1-Hus1) are more sensitive to DNA replication inhibitors, such as hydroxyurea (HU), than each single mutant [76]. These results demonstrate that DNA integrity checkpoints play an essential role in maintaining the viability of strains with circular chromosomes in the presence of DNA replication inhibitors. In other words, strains with circular chromosomes have a higher risk of genomic instability during DNA replication. These findings also demonstrate that screening of genes that genetically interact with *pot1* in the presence of DNA replication inhibitors will be useful for identifying and studying proteins involved in DNA damage checkpoint. 

Telomeres are attached to the nuclear envelope by the interaction between the nuclear envelope protein Bqt4 and the telomere-associated protein Rap1 in fission yeast [79]. When telomere-binding proteins are visualized using a fluorescent protein, one or two dots can be detected, indicating that telomeres are clustered [80]. However, the importance of telomere clustering and tethering to the nuclear envelope remains unclear. It is possible that the *pot1* disruptant, which has circular chromosomes, loses the tethering of telomeres to the nuclear envelope because more than 10 kb of the telomere ends are lost [30]. Moreover, 3D genome organization may be affected by *pot1* disruption. Therefore, screening of genes that genetically interact with *pot1* will be useful for studying the importance of telomere tethering to the nuclear envelope and telomere clustering. 

Recently, single-chromosome fission yeast models have been reported [81]. The chromosome fusions in these strains caused few defects in cell morphology and growth and had a limited impact on gene expression. As these strains have a linear chromosome, it would be interesting to compare the phenotype of the *pot1* disruptant that has a single circular chromosome with the strain that has a single liner chromosome. 

## 9. Biology of Yeast Carrying Ring Chromosomes and Comparison of the Difference between Six Types of *S. pombe* Mutants That Have Circular Chromosomes

There are six types of mutant that have circular chromosomes in *S. pombe,* namely, *pot1*/*tpz1* disruptant [22], *trt1* disruptant [25], *rad3 tel1* double mutant [31], *poz1 ccq1* double mutant [22], *ten1*/*ste1* disruptant [82], and *taz1 rad11* double mutant [83]. A common feature of these survivors is the complete loss of the telomeric repeat sequence. Sexual reproduction is severely affected with reduced spore viability in *trt1* disruptant and *rad3 tel1* double mutant [31], demonstrating that circular chromosomes without telomere sequence cause problems during meiosis in *S. pombe* [25,31,84]. 

There are similarities and differences between the six types of *S. pombe* mutant that have circular chromosomes. The *trt1* disruptant and *rad3 tel1* double mutant lose telomere gradually before chromosome circularization, while the *pot1*/*tpz1* disruptant, *poz1 ccq1* double mutant, *taz1 rad11* double mutant, and *ten1/ste1* disruptant lose telomere rapidly [22,25,31,82,83]. These facts allow us to categorize these mutants into two groups (rapid telomere loss group and gradual telomere loss group). Regarding Rad3 and Tel1 phosphorylates Ccq1 which recruit telomerase, the Trt1 recruitment is mostly eliminated in the *rad3 tel1* double mutant [85], suggesting that the mechanism of telomere loss in the *rad3 tel1* double mutant is due to a defect in the recruitment of telomerase, explaining why Rad3 and Tel1 are in the gradual telomere loss group. Pot1, Tpz1, Poz1, and Ccq1 form a protein complex to protect the telomere end [22], explaining why they are in the rapid telomere loss group. Overexpression of Pot1 suppresses rapid telomere loss in the *taz1 rad11* double mutant, suggesting that the mechanism of telomere loss in the *taz1 rad11* double mutant is related to a defect in the Pot1 function [83]. Although deletion of *stn1/ten1* also results in rapid telomere loss, the phenotype of the *stn1-1* ts mutant is frequent replication fork collapses specifically in the subtelomere regions, suggesting that the mechanism of telomere loss in *stn1* disruptant is different from that in the *pot1* disruptant [75]. 

## 10. Conclusions and Perspectives on Genes which Genetically Interact with the Telomere Protection Gene *pot1* in Fission Yeast

This review demonstrates that the screening of genes that genetically interact with fission yeast *pot1* provides many potential genes that play important roles in chromosome maintenance. Genetic interactions with *pot1* are summarized in Table 1. Proteins involved in SSA are essential for the growth of *pot1* disruptants because no chromosomal fusion would result in the extensive degradation of uncapped telomeres. The Rqh1-Top3 complex is essential for the maintenance of circular chromosomes, probably because spontaneous DNA breaks are repaired by HR, producing circular chromosome dimers that induce breakage–fusion–bridge cycles, resulting in cell death. The genes encoding CPC are also synthetically lethal with *pot1*, suggesting that the CPC is important for chromosome segregation in strains with circular chromosomes. Moreover, Bdf2 and Mst1, which are required for maintaining proper histone H4 acetylation levels, are required for the growth of cells with circular chromosomes. Changes in histone H4 acetylation levels may affect whole-genome gene expression. Therefore, the analysis of gene expression in *bdf2* and *mst1* mutants will be the next step in understanding the importance of Bdf2 and Mst1 in the growth of cells with circular chromosomes. 

A strain with a linear chromosome should deal with topological stress during DNA replication, transcription, and chromosome segregation. A strain with circular chromosomes is more sensitive to topoisomerase I and II inhibition, suggesting that circular chromosomes are topologically constrained. These results demonstrate that a strain with circular chromosomes is a useful model system for studying how cells deal with topological stress. The growth of the strain that has circular chromosomes depends on the 9-1-1 DNA damage checkpoint function when DNA replication is inhibited, suggesting that circular chromosomes are more vulnerable to replication inhibition than linear chromosomes. Finally, phosphatidylinositol 4-kinase Pik1 is essential for the growth of *pot1* disruptants, probably because of its role in the growth of cells with circular chromosomes. Although the mechanism of this finding remains unclear, our results suggest the importance of the regulation of PI(4)P levels in the Golgi and possibly in the nucleus. In addition to genetic interaction discussed here, genome-wide genetic interaction analysis for *S. pombe* revealed that *pot1* has negative genetic interactome with *atp10* and *prz1* and positive genetic interaction with SPAC227.17c [86]. However, these interactions are not studied in detail. Further research will be necessary to understand the mechanism of these genetic interactions. 

Prokaryotes such as *E. coli* have a circular chromosome. In contrast, eukaryotes, including yeasts and humans, have linear chromosomes. One evolutionary question is why natural selection chooses linear chromosomes in eukaryotes, even though linear chromosomes have DNA end replication problems. The studies presented in this review clearly demonstrate that the stable maintenance of circular chromosomes is more difficult than that of linear chromosomes during mitosis. Prokaryotes only have one circular chromosome, whereas eukaryotes have multiple linear chromosomes. The HR between sister chromatids in circular chromosomes can create circular chromosome dimers, which in *E. coli* are converted to monomers by Xer site-specific recombination [87]. The resolution of dimeric chromosomes using Xer site-specific recombination is possible for a single circular chromosome, but it may be more difficult if *E. coli* has more than two circular chromosomes. This may be the reason why prokaryotes have a single circular chromosome and it makes it difficult to increase the genome size in prokaryotes. In contrast, linear chromosomes do not require an Xer site-specific recombination system, making it possible to maintain more than two linear chromosomes without problems. This could be the reason why eukaryotes can maintain multiple linear chromosomes that can easily increase their genome size. Therefore, during evolution, eukaryotes might have acquired linear chromosomes with a telomere maintenance system before they acquire a sexual reproduction system. The importance of telomeres in meiosis also could be the reason why natural selection chooses linear chromosomes in eukaryotes, which is discussed in the review by the Ishikawa group [88]. 

In conclusion, the screening of genes that genetically interact with fission yeast *pot1* is a powerful method for studying the role of proteins required for telomere fusion, telomere degradation, and the maintenance of circular chromosomes. These studies are also informative for understanding the mechanism underlying the maintenance of linear chromosomes.

## Figures and Tables

**Table 1:** Summary of *pot1*′s genetic interactions.

Gene Name (Related Functions)	Phenotype (References)
Lethal genetic interaction with *pot1*	
*lig4*, *rad16*, *rad22*, *srs2* (Single strand annealing (SSA))	No chromosome fusion by deletion of SSA genes [30]
*rqh1*, *top3* (dissolution of double holiday junction)	Circular chromosome is not maintained by deletion of *rqh1* or mutation in *top3* [33]
*ark1*, *bir1*, *pic1* (CPC) (chromosome segregation)	Circular chromosome is not maintained by mutation of CPC [54]
*bdf2*, *mst1* (maintaining proper histone H4 acetylation level)	Circular chromosome causes growth defect by *bdf2* or *mst1* mutation [61]
*pik1* (regulation of PI(4)P level in Golgi and possibly in nucleus?)	Circular chromosome causes growth defect by *pik1* mutation? [67]
Suppresion of *pot1*, *pot1 rqh1* double and *pot1 rqh1-hd* double mutant phenotype	
*rqh1* (Helicase)	*rqh1-hd* mutation rescues telomere loss of *pot1*Δ cells [45]
*chk1*, *wee1 mik1* double mutation, *cdc2-3w* (G2 length regulation)	*chk1* or *cdc2-3w* mutation, or *wee1 mik1* double mutation rescue TBZ sensitivity of *pot1 rqh1-hd* cells [47,48]
*rad51*, *exo1 rqh1* double mutation (HR)	Lethality of *pot1 rqh1* is suppressed by deletion of *rad51* or *exo1* [33]
*exo1*, *rqh1* (processing of uncapped telomere)	Acute growth defect by *pot1* shut-off is suppressed by *exo1* and/or *rqh1* mutation [32]
Enhancement of *pot1* and *pot1 rqh1* double mutant phenotype	
*bub1*, *mad2* (Spindle checkpoint)	Growth of *pot1 rqh1-hd* cells becomes worse by *bub1* or *mad2* mutation [47]
*hus1*, *rad1*, *rad9* (9-1-1 complex) (DNA damage checkpoint)	Mutation in 9-1-1 complex increases HU sensitivity of *pot1*Δ cells [76]
*swi6* (Heterochromatin), *sgo2* (Sister chromatid biorientation)	Mutation of *swi6* or *sgo2* increases chromosome segregation defect of *pot1*Δ cells [54]
Phenotype suppression by deletion of *pot1*	
*dcr1* (RNA interference machinery)	Pericentric silencing defect in *dcr1*Δ cells is suppressed by *pot1* deletion [58]

## Data Availability

Not applicable.
